# Overexpression of Brain- and Glial Cell Line-Derived Neurotrophic Factors Is Neuroprotective in an Animal Model of Acute Hypobaric Hypoxia

**DOI:** 10.3390/ijms23179733

**Published:** 2022-08-27

**Authors:** Maria S. Gavrish, Mark D. Urazov, Tatiana A. Mishchenko, Victoria D. Turubanova, Ekaterina A. Epifanova, Victoria G. Krut’, Alexey A. Babaev, Maria V. Vedunova, Elena V. Mitroshina

**Affiliations:** 1 Institute of Biology and Biomedicine, Lobachevsky State University of Nizhny Novgorod, 23 Gagarina Ave., 603022 Nizhny Novgorod, Russia; 2 Institute of Cell Biology and Neurobiology, Charité-Universitätsmedizin Berlin, Charitéplatz 1, 10117 Berlin, Germany; 3 Scientific Center for Genetics and Life Sciences, Sirius University of Science and Technology, 354340 Sochi, Russia

**Keywords:** hypoxia, brain-derived neurotrophic factor, BDNF, glial cell-derived neurotrophic factor, GDNF, adeno-associated viral vector, neuroprotection

## Abstract

Currently, the role of the neurotrophic factors BDNF and GDNF in maintaining the brain’s resistance to the damaging effects of hypoxia and functional recovery of neural networks after exposure to damaging factors are actively studied. The assessment of the effect of an increase in the level of these neurotrophic factors in brain tissues using genetic engineering methods on the resistance of laboratory animals to hypoxia may pave the way for the future clinical use of neurotrophic factors BDNF and GDNF in the treatment of hypoxic damage. This study aimed to evaluate the antihypoxic and neuroprotective properties of BDNF and GDNF expression level increase using adeno-associated viral vectors in modeling hypoxia in vivo. To achieve overexpression of neurotrophic factors in the central nervous system’s cells, viral constructs were injected into the brain ventricles of newborn male C57Bl6 (P0) mice. Acute hypobaric hypoxia was modeled on the 30th day after the injection of viral vectors. Survival, cognitive, and mnestic functions in the late post-hypoxic period were tested. Evaluation of growth and weight characteristics and the neurological status of animals showed that the overexpression of neurotrophic factors does not affect the development of mice. It was found that the use of adeno-associated viral vectors increased the survival rate of male mice under hypoxic conditions. The present study indicates that the neurotrophic factors’ overexpression, induced by the specially developed viral constructs carrying the BDNF and GDNF genes, is a prospective neuroprotection method, increasing the survival rate of animals after hypoxic injury.

## 1. Introduction

Hypoxia is one of the key damaging factors involved in the development of many brain pathologies, including ischemic stroke, neurodegenerative diseases, and brain tumors. So far, there are no effective methods for correcting the consequences of hypoxic central nervous system (CNS) damage. The most promising therapeutic approach is the creation of a set of procedures based on the use of endogenous regulatory molecules with a pronounced neuroprotective effect. Exogenous or endogenous stimulation of these regulatory systems will make it possible to maintain cell viability under stress conditions and launch adaptive mechanisms that preserve their functional activity.

The neurotrophic factors BDNF and GDNF are important signaling molecules that contribute to maintaining the functional integrity and survival of neurons under stress conditions [1,2]. Numerous experimental studies have shown their effectiveness in modeling various pathologies of the brain, including hypoxic conditions and neurodegenerative diseases [3,4,5,6,7]. Therefore, these neurotrophic factors can be considered potential therapeutic agents for the correction of the central nervous system’s most complex pathologies, which often lead to the death or disability of the patient.

Possibility of long-term increase of endogenous neurotrophic factors expression level using genetic engineering methods is of particular interest [8]. These technologies allow solving the problem of a long-term stable increase in the target compounds level, overcoming the blood–brain barrier, as well as achieving expression in specific populations of nerve cells. Adeno-associated viruses (AAV) are a type of viral vectors widely used to deliver genes of interest to the nervous system’s cells. The AAV attractiveness lies in the absence of pathogenicity, ensuring the stable long-term expression of target genes and the possibility of transducing both dividing and non-dividing cells, which is especially important for their use in nervous tissue [9]. Moreover, in vitro studies have shown that BDNF overexpression prevents neuronal apoptosis in neurodegenerative diseases [10] and maintains the complex structure of neural networks in the post-hypoxic period, which allows partially leveling the negative effects of hypoxia, maintaining functional neural network activity [6,11]. A number of studies using experimental models of ischemic stroke in vivo confirm the effectiveness of using viral constructs carrying neurotrophic factors’ genes. For instance, AAV-mediated delivery of BDNF to the striatum 4–5 weeks before modeling ischemia reduced the death of rat brain neurons in a transient model of middle cerebral artery occlusion [12]. Overexpression of BDNF through AAV-BDNF gene therapy promoted the migration of endogenous neuronal progenitor cells from the subventricular zone, which stimulated animals’ functional recovery after brain ischemia modeling [13]. Delivery of the GDNF gene reduced the death of neurons in the cerebral cortex in ischemia by preventing apoptotic processes activation [14,15]. It was also shown that BDNF gene therapy using lentiviral constructs improved the indicators of animals’ motor activity and emotional state in the postischemic period [16].

Nevertheless, some studies cast doubt on gene therapy’s effectiveness based on a long-term increase in the level of neurotrophic factors’ production. For example, Arvidsson et al. showed that the AAV-mediated overexpression of GDNF does not protect striatal neurons after the middle cerebral artery occlusion and, in fact, exacerbates ischemia-induced neuronal loss without contributing to the restoration of nerve cells functional activity [17]. Gustafsson et al. demonstrated that high levels of BDNF expression, induced by retrograde axonal AAV transport, increase the vulnerability of striatal interneurons to ischemic damage [18]. The contradictory results point to the need for further studies of the genetic constructs’ action mechanisms on morpho-functional changes in the nervous system’s cells in normal and hypoxic/ischemic conditions.

In addition, earlier, using recombinant proteins BDNF and GDNF, we showed that the use of both BDNF and GDNF can significantly correct the negative consequences of hypoxic brain damage, but their combined application is less effective than the use of each of these neurotrophic factors separately [19]. When investigating a possible molecular mechanism underlying this effect, we showed that the exogenous application of the neurotrophic factors BDNF and GDNF in the form of recombinant proteins under normoxia affects the level of BDNF mRNA expression in primary hippocampal cell cultures. BDNF application stimulated BDNF mRNA synthesis in cells of primary hippocampal cultures; GDNF application resulted in suppression of BDNF mRNA expression, which was more pronounced in neurons than in astrocytes [20]. This may be a limiting factor in the chronic application of neurotrophins for the correction of hypoxic brain damage. Therefore, in this work, we wanted to determine whether the overexpression of each of the studied neurotrophins affects the production of another neurotrophic factor, as well as their key receptors.

Thus, the present study aimed to evaluate the antihypoxic and neuroprotective properties of BDNF and GDNF expression level increase using adeno-assisted viral vectors in modeling hypoxia in vivo. Moreover, we aimed to determine whether the overexpression of each of the studied neurotrophins affects the production of another neurotrophic factor, as well as their key receptors.

## 2. Results

To achieve an aim of a long-term increase in the production of neurotrophic factors BDNF and GDNF by brain cells through the use of genetic constructs, we performed an intraventricular injection of the developed original viral vectors AAV-Syn-BDNF-eGFP and AAV-Syn-GDNF-eGFP to the newborn mice (P0).

**Figure 1:**
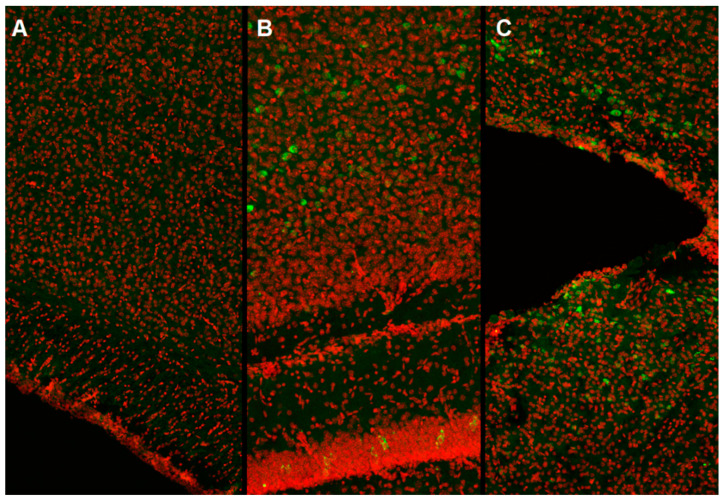
Representative confocal images of eGFP fluorescent protein expression in mouse brain slices after transduction with the AAV-vector: (**A**)—Intact, (**B**)—AAV-Syn-BDNF-eGFP, (**C**)—AAV-Syn-GDNF-eGFP. Red—DAPI, Green—eGFP. Image size 640 × 1920 µm. A laser scanning microscope LSM 800, Plan-Apochromat 10×/0.3 objective (Zeiss, Oberkochen, Germany) was used for the imaging.

In our work, we used the method of intracerebroventricular injection of a viral vector in newborn male C57BL/6 mice (P0). It was found that starting from the third week of postnatal development, stable expression of the green fluorescent protein, which is part of the vector, is achieved (Figure 1).

**Figure 2:**
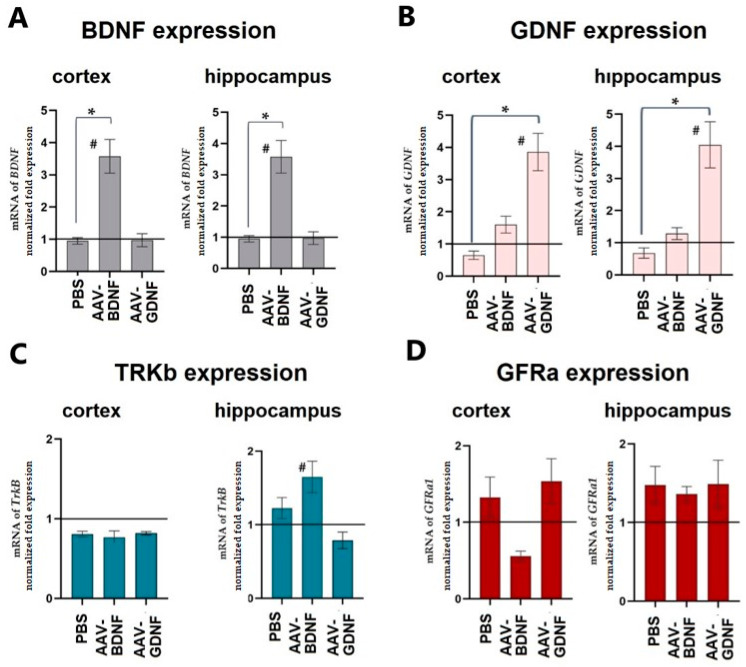
Expression levels of the neurotrophic factors BDNF (**A**), GDNF (**B**) and their receptors–TRKb (**C**) and GFRa (**D**), in the tissues of the cerebral cortex and hippocampus on day 30 after viral transduction (P30). Data were normalized relative to intact animals. #—statistically significant difference compared to the“PBS” group), *—statistically significant difference between experimental groups, *p* ≤ 0.05 (one-way ANOVA and Tukey’s multiple post-hoc test).

The level of neurotrophic factor expression was assessed on day 30 after expression in the cerebral cortex and hippocampus. The adeno-associated vector constructs developed by the authors were shown to effectively increase the production of the target neurotrophic factor in both studied brain regions. Upon AAV-Syn-BDNF-eGFP tissue transduction, BDNF production increased on P30 3.39 ± 0.42-fold in the cerebral cortex and 3.57 ± 0.56-fold in the hippocampus (Figure 2). PBS injection did not alter the production of neurotrophic factors. We also verified that the AAV-Syn-BDNF-eGFP virus did not alter GDNF production. Interestingly, an increase in the expression of a brain-derived neurotrophic factor in the hippocampus increased the expression level of TRKb receptors by 1.65 ± 0.18 times. At the same time, no changes in the level of TRKb expression were observed in the cerebral cortex.

Assessment of GDNF expression levels showed that transduction of AAV-Syn-GDNF-eGFP resulted in a 3.85 ± 0.61-fold increase in GDNF mRNA levels in the cortex and a 4.04 ± 0.68-fold increase in the hippocampus. GNDF production did not change in the groups with the intraventricular injection of PBS and AAV-Syn-BDNF-eGFP. No significant changes in the expression of the GDNF receptor GFRa were found. Thus, the application of the adeno-associated viral constructs carrying the genes of neurotrophic factors developed by us led to a persistent increase in their expression.

**Figure 3:**
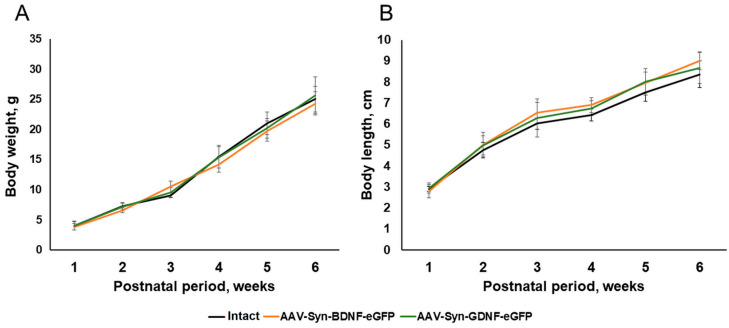
Dynamics of changes in morphometric parameters of male C57BL/6 mice after AAV-Syn-BDNF-eGFP and AAV-Syn-GDNF-eGFP transduction. (**A**) the dynamics of changes in the weight of mice within six weeks after the injection of the AAV-constructs; (**B**) the dynamics of changes in the body length of mice within six weeks after the injection of the AAV-constructs. No statistically significant differences between groups.

To determine the possible effects of the AAV-Syn-BDNF-eGFP viral vectors on the development of newborns in the postnatal period, we analyzed the dynamics of changes in the weight and growth characteristics of mice within six weeks after the injection of the viral preparation. As a result of the studies, it was shown that the overexpression of neurotrophic factors did not affect the morphometric parameters of animals. The appearance of mice of the AAV-Syn-BDNF-eGFP and AAV-Syn-GDNF-eGFP groups, their weight, and growth parameters corresponded to the norm. No significant differences with the “Intact” group were found during the entire observation period (Figure 3).

Neurotrophic factors are involved in the regulation of nerve cell differentiation, synaptogenesis, and other important processes associated with brain formation. In our experiment, the introduction of viral vectors encoding BDNF and GDNF was carried out in newborn animals. Therefore, we assessed the potential effect of BDNF and GDNF hyperexpression on the state of the nervous system in mice. For this purpose, the neurological status of the animals on P30 was assessed according to the scale for assessing the neurological deficit in small laboratory animals and the Garcia scale, the results of which are presented in Table 1 of the manuscript. No neurological abnormalities were detected. The development of the animals corresponded to their age (Table 1).

**Figure 4:**
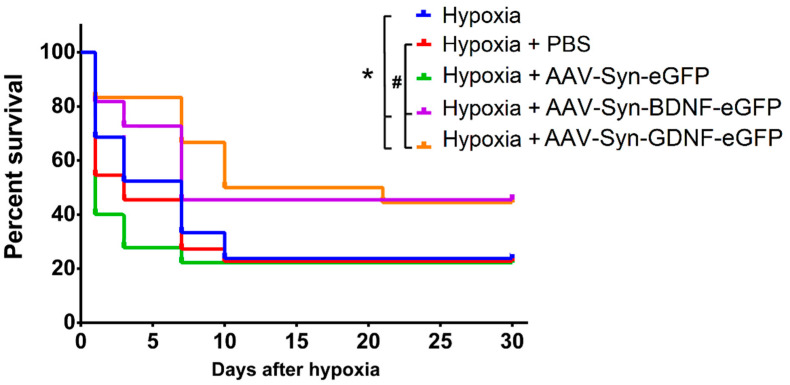
Survival rate of male C57BL/6 mice after simulating acute hypobaric hypoxia amid preventive administration of AAV-Syn-BDNF-eGFP and AAV-Syn-GDNF-eGFP viral vectors (“Hypoxia” n = 21; “Hypoxia + PBS” n = 22; “Hypoxia + AAV-Syn-eGFP” n = 18; “Hypoxia + AAV-Syn-BDNF” n = 22; “Hypoxia + AAV-Syn-GDNF” n = 18). The statistical difference from “Hypoxia” (*) or “Hypoxia + PBS” (#) was calculated by a long-rank Mantel-Cox test, *, # *p*  <  0.05.

To assess the neuroprotective effects of the neurotrophic factors BDNF and GDNF overexpression, the animals were exposed to acute hypobaric hypoxia on day 30 after transduction (P30). It was found that transduction by both the AAV-Syn-BDNF-eGFP and AAV-Syn-GDNF-eGFP vectors increases the resistance of animals to hypoxic damage in the late post-hypoxic period. Then, 24 h after the simulated stress, animals’ survival rate in the experimental (“Hypoxia + AAV-Syn-BDNF-eGFP” and “Hypoxia + AAV-Syn-GDNF-eGFP”)) and control groups (“Hypoxia”, “Hypoxia + PBS”, “Hypoxia + AAV-Syn-eGFP”) did not differ significantly (Figure 4). However, the survival rate of animals with overexpression of both neurotrophic factors in the late post-hypoxic period (30 days after AHH) was significantly higher than in all control groups. In groups “Hypoxia + AAV-BDNF” and “Hypoxia + AAV-GDNF”, the survival rate was 44% and 45%, and in “Hypoxia no injection”, “Hypoxia + PBS”, and “Hypoxia + AAV-Syn-eGFP” was 24%, 23%, and 27%, respectively.

**Figure 5:**
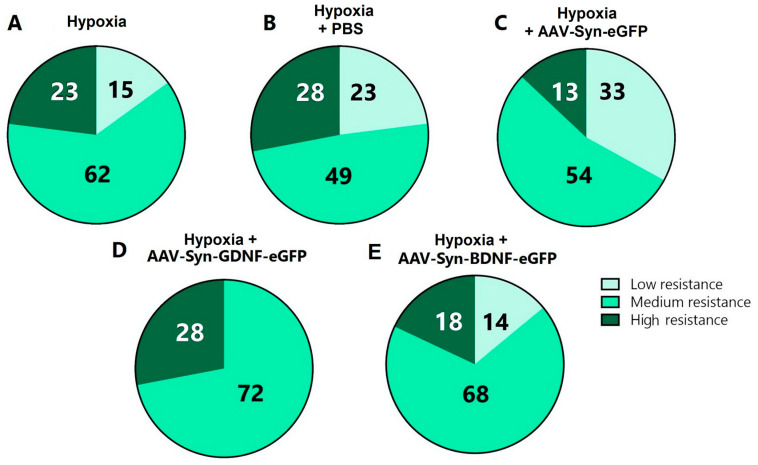
Distribution of animals according to the degree of resistance to acute hypobaric hypoxia, %. (**A**)—“Hypoxia”; (**B**)—“Hypoxia + PBS”; (**C**)—“Hypoxia + AAV-Syn-eGFP; (**D**)—“Hypoxia + AVV-Syn-GDNF-eGFP”; (**E**)—“Hypoxia + AAV-Syn-GDNF-eGFP”. Animals with a survival time of less than 3 min were classified as low-resistant, from 3 to 7 min–as medium-resistant, and more than 7 min–as highly resistant.

It was also revealed that transduction by the AAV-Syn-GDNF-eGFP vector increases the animals’ resistance to hypoxia. Compared with the “Hypoxia” group (low resistance 15%, medium resistance 62%, high resistance 23%), “Hypoxia + PBS” group (low resistance 23%, medium resistance 49%, high resistance 28%), and “Hypoxia + AAV-Syn-eGFP” group (low resistance 33%, medium resistance 54%, high resistance 13%), the share of moderately resistant animals in “Hypoxia + AAV-Syn-GDNF” group was higher (72%, *p* ≤ 0.05). There were no low-resistant animals among those featuring overexpression of the glial cell-derived neurotrophic factor (Figure 5).

In the next step, we conducted an immunocytochemical analysis of brain tissues after hypoxic injury. ICC was carried out on day 1 and 7 days after hypoxia. Neurons were labeled with antibodies to NeuN and MAP2, as well as astrocytes with anti-GFAP.

**Figure 6:**
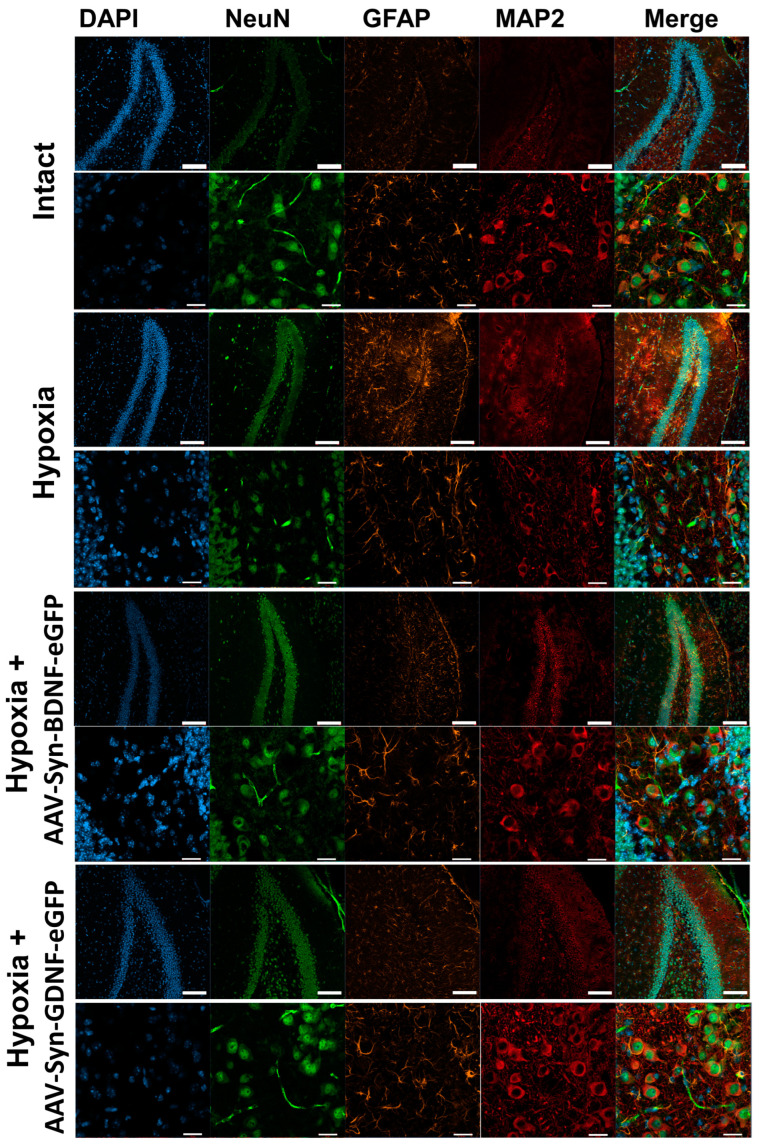
Representative confocal images of ICC of mouse brain slices on day 7 after acute hypobaric hypoxia modelling: “Intact”, “Hypoxia”, “Hypoxia + AAV-Syn-BDNF-eGFP”,”Hypoxia + AAV-Syn-GDNF-eGFP”. Blue—DAPI, Green—NeuN, Orange—GFAP, Red—MAP2. Odd rows-scale bar–100 µm (Plan-Apochromat 10×/0.3 objective). Even rows -scale bar–20 µm Plan-NeoFluar 40×/0.75 objective. A laser scanning microscope LSM 800 (Zeiss, Oberkochen, Germany) was used for the imaging.

On day 1 after modeling hypoxia, no pronounced changes in the cerebral cortex and hippocampus were detected. On day 7, the samples of mice from the “Hypoxia” group showed changes in the morphology of neurons: fragmentation of processes and an increase in the size of neuronal bodies (Figure 6). Slight astrogliosis was also noted, as the number of GFAP+ astrocytes in the hippocampus was increased.

In the brain tissues of mice from the “Hypoxia + AAV-Syn-BDNF-eGFP” and “Hypoxia + AAV-Syn-GDNF-eGFP” groups, no changes in the morphology of neurons and astrocytes were detected.

**Figure 7:**
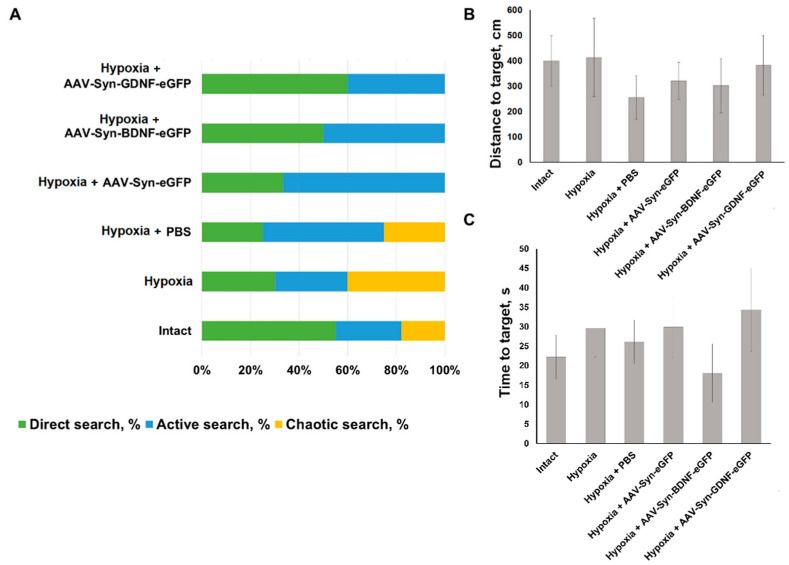
(**A**–**C**) Distribution of target search strategies, time and distance to target of animals tested in the Morris water maze, %. No significant differences between the different groups were found, *p* < 0.05, the Kruskal–Wallis test.

To assess the effect of neurotrophic factors overexpression on animals’ mnestic and cognitive abilities in the late post-hypoxic period, testing in the Morris water maze was carried out. The learning protocol includes five sessions of three attempts each. All the animals of the intact group have found the hidden platform in the third learning session. In the “Hypoxia” group, two animals have failed to find the hidden platform on the third learning session. One animal was noted to have one failed attempt of three on the fifth learning session. In “Hypoxia + PBS” group one animal has failed one attempt to find the hidden platform on the third learning session and one attempt to find the hidden platform on the fifth learning session. In groups with intraventricular viral vectors injection no failed attempts were detected on the fifth session. When evaluating the reproduction of a memory trace, a change in the distribution of target search strategies was observed. In the “Hypoxia” group, 40% animals demonstrated a chaotic search for a maze platform. In animals with overexpression of the neurotrophic factor BDNF, target search strategies were divided evenly between direct (direct movement to the target) and active search (radial search movements along the maze perimeter before reaching the target). In the group Hypoxia with overexpression of the neurotrophic factor GDNF, 60% of the animals chose direct target search, 40%–active one (Figure 7). However, no significant differences between the delayed coefficient of long-term memory retention in different animals’ groups were found. For the “Intact” group, its value was 40.5 ± 7.5%, “Hypoxia” group value was 36.12 ± 10.04%, for animals in the “Hypoxia + PBS” group 43.49 ± 9.720%, “Hypoxia + AAV-Syn-eGFP” 32.74 ± 9.91%, “Hypoxia + AAV-SynBDNF-eGFP” 49.36 ± 10.20%, and “Hypoxia + AAV-Syn-GDNF-eGFP” 38.86 ± 6.517%. Moreover, no significant differences between the distance and time to target in different animals’ groups were found (Figure 7).

## 3. Discussion

Neurotrophic factors BDNF and GDNF are important signal molecules that take part in neurogenesis regulation, neuronal growth and survivability. BDNF is known to modulate synaptic transmission in mature brain. BDNF is synthetized mostly by neurons and glial cells, but could be produced by endothelium cells, immune cells, etc. [21]. It is expression was observed in hippocampus, frontal cortex, midbrain, amygdala, hypothalamus, striatum, pons, and medulla. In both normal and ischemic/hypoxic conditions, BDNF protective action is mediated by interaction with tyrosine receptor kinase B (TrkB) and launch of the main metabolic signaling cascades [22,23]. The member of the transforming growth factor-beta superfamily neurotrophic factor GDNF promotes the maintenance, proliferation, and differentiation of cell populations in the central and peripheral nervous system. GDNF is shown to be one of the main factors produced by glial cells for neuronal viability in stress conditions and plays important neuroprotective role in neurodegenerative disease [24,25].

Both BDNF and GDNF are known for its neuroprotective effects in different pathologies of central and peripheral nervous systems, including hypoxic and ischemic damage [2,4,26], traumas, neurodegenerative diseases, Parkinson disease [27,28], and Alzheimer disease [29,30]. The main effect of BDNF and GDNF is related to receptor-mediated launch of metabolic cascades that includes different kinases and activation of many branches of that cascades support cell viability [26,29,31]. It was demonstrated that the level of production of neurotrophic factors decreases with the development of neurodegenerative processes. Interestingly, activation of the TrkB receptor by a CF3CN agonist significantly increases BDNF levels in 3xTg mice [29]. One of the recently described pathways for BDNF level regulation is the conserved Wnt signaling pathway, which is involved in the regulation of neuronal cells proliferation and differentiation. For example, a seven-day administration of Wnt-3a increased BDNF expression, proliferation, and migration of neuroblasts from the subventricular zone after ischemia modeling [32], while the inhibitor of the Wnt/β-catenin pathway suppressed the expression of Wnt3a, nuclear β-catenin, BDNF, and MBP, and worsened the neurological status of animals after stroke [33].

Indeed, a number of studies have shown that recombinant neurotrophic factors are highly effective in models of various pathologies, including cerebral ischemia and neurodegenerative diseases, both in vitro and in vivo [25,26,34,35,36]. However, many methodological difficulties, including overcoming the blood–brain barrier and the short period of active molecules’ degradation, call into question the potential use of BDNF and GDNF in clinical practice. Gene therapy with viral vectors can overcome these limitations and provide sustained overexpression of BDNF and GDNF in the nervous system’s tissues, where it is necessary to maintain neuronal survival.

To date, there are still very few studies on the effectiveness of gene therapy based on vectors carrying genes for neurotrophic factors. Therefore, the evaluation of the effects of using adeno-associated viral vectors carrying the BDNF or GDNF gene in various CNS pathologies is highly relevant. There are a number of papers devoted to the protective effect of these vectors in various neurodegenerative diseases, such as retinal neurodegeneration, Alzheimer’s disease, Parkinson’s disease, and others [37,38,39,40]. However, studies on the possibility of using genetic engineering approaches that induce overexpression of neurotrophic factors to correct ischemic damage have just begun to emerge. For example, Ahn et al. have shown that BDNF-overexpressing engineered human mesenchymal stem cells can correct hypoxia-induced disorders several times more effectively than naive stem cells both in vitro and in vivo [41].

There are only a few research on the protective effect of viral vectors carrying genes for neurotrophic factors in modeling ischemia or hypoxia. For example, Yu et al. [13] showed that when a viral preparation was injected into the subventricular zone of adult rats 14 days before the right middle cerebral artery occlusion, the animals recovered their motor activity faster. A recent study by Xue et al. demonstrated that overexpression of BDNF using an HSV vector can promote axonal regeneration and prevent neuronal death in a model of glucose-oxygen deprivation [42].

The information on the effectiveness of genetic engineering approaches that induce GDNF overexpression for the correction of ischemic-hypoxic damage is even more limited. In Markosyan et al., 2020 [43], the use of adeno-associated vectors carrying the VEGF, GDNF, and NCAM genes reduced infarct size in distal middle cerebral artery occlusion in rats. Another study notes the positive effect of the use of the lentiviral construct Lv-GDNF in middle cerebral artery occlusion in mice, which manifested itself in a decrease in the number of degenerated neurons in the striatum [44].

To sum up, to date, there are very few studies that cover the effect of BDNF and GDNF overexpression on the resistance of animals to hypoxic damage, and there are no works on the mutual influence of the overexpression of neurotrophic factors BDNF and GDNF, which makes our study demanded and relevant.

In our work, we used the method of intracerebroventricular injection of a viral vector in newborn male C57BL/6 mice (P0). Previously published studies, in which the viral construct AAV-Syn-BDNF-eGFP developed by us was used, demonstrated not only the therapeutic effect under the conditions of hypoxia, oxygen-glucose deprivation (OGD) and glutamate-induced excitotoxicity but also the fact that the eGFP fluorescent label did not obstruct the BDNF release [6,34]. Staining of presynaptic secretory granules of neurons with a fluorescent probe FM-4-64 also shows that BDNF together with eGFP are localized in secretory granules. A significant decrease in eGFP fluorescence was observed several minutes after OGD induction, which indicated the BDNF release and coincided with the first (reversible) phase of the OGD-induced increase in [Ca^2+^]. Consequently, the BDNF release occurs during the initial phase of OGD, which may contribute to the rapid effects of BDNF in neuroglial networks [34].

Our previous study indicates that eGFP does not interfere with the packing of BDNF linked with it into presynaptic secretory granules, does not hinder release, and that the release of BDNF-eGFP is a Ca^2+^-dependent process [34]. The present manuscript focuses on studying the effects of the use of adeno-associated viral vectors carrying the genes of the neurotrophic factors BDNF and GDNF for the improvement of the resistance of animals to hypoxia. Viral constructs were injected into the brain ventricles of newborn C57Bl6 (P0) mice. In the present work, we carefully investigated the potential mutual influence of neurotrophins in the application of viral vectors. However, we did not find any cross interaction. The adeno-associated vectors developed by the authors were shown to effectively increase the production of only the target vector-encoded neurotrophic factor. We examined the expression of neurotrophins in two brain regions (the sensorimotor cortex and the hippocampus). The level of expression of neurotrophic factors was assessed at P30 before modeling acute hypobaric hypoxia.

The study of downstream BDNF and GDNF cascades, such as TrkB and RET-GFRa, is of great interest. Previously, we devoted a series of works to this topic. For example, in [4], we used primary neuronal cultures to show that the protective effects of BDNF are associated with the activation of the TrkB receptor. For this purpose, we used K252a, a tropomyosin receptor kinase B (TrkB) antagonist. The blockade of TrkB with K252a completely abolished the supporting effect of neurotrophin both on the viability of nerve cells and on their bioelectrical and calcium spontaneous activity.

In addition, through the chronic application of ANA-12, the more highly selective TrkB antagonist, we showed that TrkB receptor activity is necessary for the formation of a full-fledged spontaneous neural network burst bioelectrical activity [45]. ANA-12 binds specifically to the TrkB-d5 subdomain in a dose-dependent manner and prevents BDNF-induced TrkB activation. Electron microscopy also showed that blocking TrkB receptors affects the structure of synapses (e.g., causes the reduction in size or absence of postsynaptic density (PSD) in the synapse) and the mitochondrial apparatus.

In Mitroshina et al., 2019 [2], we convincingly demonstrated that RET kinase, a component of the receptor complex, and the PI3K/Akt pathway are critical for the neuroprotective action of GDNF. The application of the RET inhibitor significantly reduced the cell viability of primary hippocampal cultures under normal conditions by 7.15% after 24 h and by 10.7% after seven days. The blockade of RET kinase completely eliminated the neuroprotective effect of GDNF during hypoxia modeling and aggravated its destructive effect on the functioning of neural networks in vitro.

To sum up, in our previously published works, we convincingly demonstrated that the protective effects of the neurotrophic factors BDNF and GDNF are mediated by activation of the TrkB and RET-GFRa receptors, respectively.

Evaluation of growth and weight characteristics and neurological status of animals showed that the overexpression of neurotrophic factors does not affect the development of mice.

Acute hypobaric hypoxia was modeled on day 30 after the injection of viral vectors. It was found that the use of AAV vectors increased the survival rate of animals under hypoxic conditions and contributed to the preservation of long-term spatial memory in the late post-hypoxic period. The use of both AAV-Syn-BDNF-eGFP and AAV-Syn-GDNF-eGFP increased the survival rate of animals in the post-hypoxic period for 30 days after modeling acute hypobaric hypoxia. It is important to note that among the animals with the intraventricular injection of AAV-Syn-GDNF-eGFP, there were no animals with a low resistance to hypoxia, i.e., the survival time at altitude was at least 3 min.

Despite the absence of pronounced memory impairments in the late post-hypoxic period, it is important to note that animals with increased expression of both BDNF and GDNF lacked the strategy of chaotic search in the Morris maze, which indicates an improvement in cognitive and mnestic functions.

It is assumed that overexpression of the neurotrophic factors BDNF, GDNF through viral constructs can be considered an effective method of neuroprotection in hypoxic states. Hypoxia can be not only an isolated pathology but also be associated with a variety of CNS diseases, such as Alzheimer’s disease, Parkinson’s disease, etc. [46,47]. Therefore, approaches that increase the brain’s resistance to hypoxia are of great practical value.

Interestingly, BDNF overexpression led to an increase in TRKb receptor expression in the hippocampus but not in the cerebral cortex. This may be one of the molecular mechanisms of the protective effect of BDNF. The specificity of the revealed effect for the hippocampus may become a subject for further research.

The present study indicates that the neurotrophic factors’ overexpression, induced by the specially developed viral constructs carrying the BDNF and GDNF genes, is an effective neuroprotection method, increasing the survival rate of animals after hypoxic injury. It is also important to note that the animals who received an injection of developed viral constructs demonstrate normal cognitive and mnestic abilities.

## 4. Materials and Methods

### 4.1. Ethics Statement

C57BL/6 newborn male mice (P1) served as the material for the research. The experimental animals were housed for 40 subsequent days in a certified SPF vivarium of the Lobachevsky State University of Nizhny Novgorod. All experimental procedures were carried out in accordance with Act 708n (23 082010) of the Russian Federation National Ministry of Public Health, which states the rules of laboratory practice for the care and use of laboratory animals, and Council Directive 2010/63 EU of the European Parliament (22 September 2010) on the protection of animals used for scientific purposes. and were approved by the Bioethics Committee of Lobachevsky State University of Nizhny Novgorod (protocol No. 14 dated 01.19.2018).

### 4.2. Development of Adeno-Associated Viral Vectors Carrying Genes of Neurotrophic Factors BDNF and GDNF

To ensure the neurotrophic factors BDNF and GDNF overexpression, adeno-associated viral vectors carrying the corresponding genes were constructed. The construction of an adeno-associated viral vector encoding the BDNF gene was carried out according to [6]. The basis for the viral vectors’ development was the AAV-Syn-eGFP-kid2 plasmid carrying the following sequences: (1) the human synapsin neuronal promoter (hSyn); (2) a WPRE regulatory element that enhances hSyn performance; (3) a multilinker for ORF cloning of the embedded gene; (4) SV40 polyA signal sequence flanked by ITR repeats from AAV serotype 2; (5) the eGFP gene. The human synapsin promoter (hSyn) was used in the viral vectors; it allows specific expression of the gene of interest in neurons [48].

**Figure 8:**
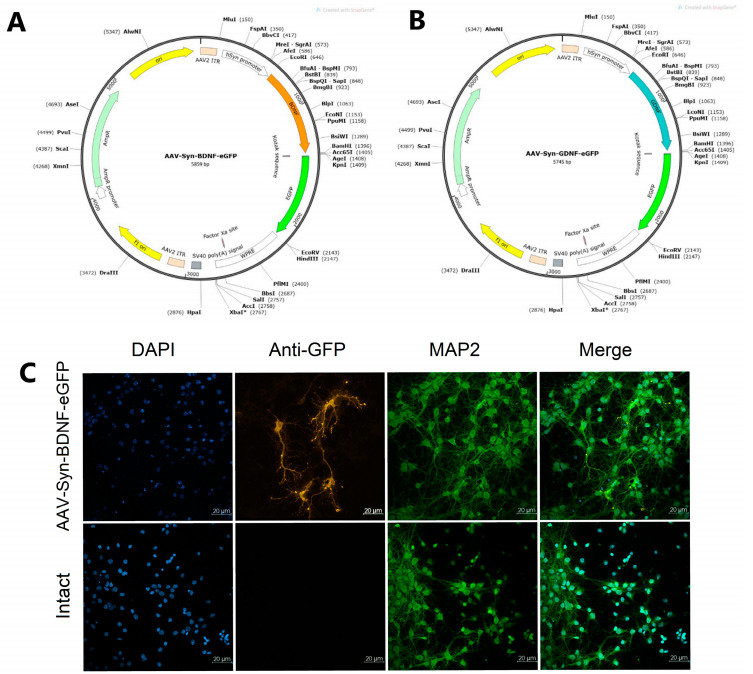
Development of adeno-associated viral vectors carrying genes of neurotrophic factors BDNF and GDNF. Maps of pAAV-Syn-BDNF-eGFP (**A**) and pAAV-Syn-GDNF-eGFP (**B**) plasmid vectors (constructed using the SnapGene Viewer 3.1.2). Immunocytochemical staining of primary cultures of mouse cortical neurons with anti eGFP, antiMAP2, and DAPI antibodies (**C**). Images were obtained using an LSM 800 laser scanning microscope, magnification ×20 (Zeiss, Oberkochen, Germany). “Intact” –non-transfected culture; pAAV-Syn-BDNF-eGFP–culture transfected with plasmid vector. (pAAV-Syn-GDNF-eGFP has a similar vector backbone and was not presented).

For the amplification and cloning of the BDNF and GDNF genes into the constructed plasmid vectors (Figure 8, AAV-Syn-BDNF-eGFP (A) and AAV-Syn-GDNF-eGFP (B)), the corresponding original primer systems were developed: mBDNF-EcoRI-fw и (5′-ATTGAATTCATGGGCCACATGCTGTCC-3′) and mBDNF-BamHI-rv (5′-AATGGATCCAATCTTCCCCTTTTAATGGTCAGTG-3′); mGDNF-NheI-fw (5′-AATGCTAGCGTCCACCATGGGATTCG-3′) and mGDNF-AgeI-rv (5′-AATACCGGTGAGATACATCCACACCGTTTAGC-3′) [6,34,49,50,51]. The viral vectors were assembled in HEK 293FT cells using the helper plasmids AAV-DJ and pHelper.

Cell type specificity after transfection pAAV-Syn-BDNF-eGFP (pAAV-Syn-GDNF-eGFP has a similar vector backbone) was detected using immunocytochemical analysis by checking the colocalization of the fluorescent protein of the virus with MAP2, a marker of differentiated neurons (Figure 8C).

### 4.3. Injection of Adeno-Associated Viral Vectors to Experimental Animals

**Figure 9:**
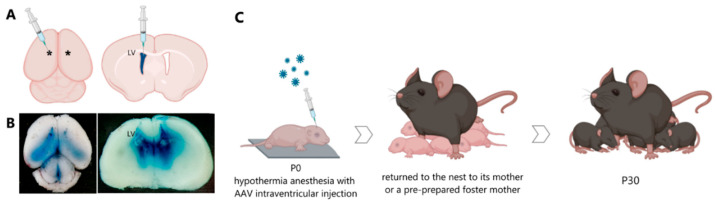
AAV intraventricular injection of neonatal mice (P0). Diagrammatic view of mouse brain shows the target sites (*) which were used for viral injection (**A**). Neonatal whole brain with coronal cross-section of brain harvested immediately after bilateral dye injection to illustrate the extent and localization of the fill (**B**). Scheme of AAV intraventricular injection (**C**). Created in BioRender.com (accessed on 26 May 2022).

In the first hours of life, newborn male mice were injected with the viral constructs AAV-Syn-BDNF-eGFP and AAV-Syn-GDNF-eGFP (Figure 9). The injections were made into the lateral ventricles of the brain at the rate of 1.7 μL per hemisphere. Trypan blue dye at a final concentration of 0.05% was used to visualize the injection of viral particles. The injection was performed with a 10 µL injection syringe with a 32 G needle. The maximum volume allowed for injection into the lateral ventricles did not exceed 2 μL. The manipulations were carried out in a sterile room in compliance with all the requirements for surgical interventions. The newborn mouse was placed on a metal plate, pre-cooled on ice, and covered with a napkin to induce hypothermia anesthesia. After a while, the mouse was checked for motor activity to confirm the effect of anesthesia. The injection was performed without the use of stereotactic equipment at one of the preselected sites: 0.8–1 mm lateral from the sagittal suture, halfway between lambda and bregma. The alternative site was located at two fifths of the distance from the lambda suture to each eye to a depth of no more than 3 mm in both cases. The injection site was marked on the newborn’s skin with a non-toxic laboratory marker. A notch was made on the needle to determine the depth of injection of the viral particle solution. The syringe with the needle was placed strictly perpendicular to the injection site, and when the plunger was pressed, the needle should not go deeper than the specified value. When all the above requirements were met, the cerebral ventricle was stained blue. The injection into the contralateral ventricle was performed in the same manner.

**Figure 10:**
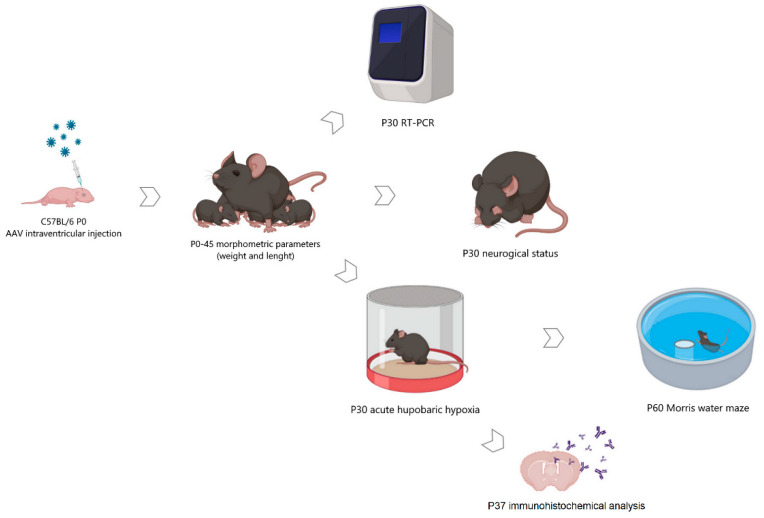
Scheme of experiments. Created in BioRender.com (accessed on 26 May 2022).

After manipulations, the mouse was marked with a non-toxic laboratory marker and placed on a warm substrate until the skin color normalized, motor and respiratory activity restored, and later returned to the nest to its mother or a pre-prepared foster mother [9,52,53] (Figure 10).

### 4.4. Brain Slices Preparation

To confirm the efficiency of transduction by the developed constructs, the eGFP protein fluorescence in the brain tissues of animals was assessed in 3 weeks after the injection of the viral preparation. In the experiments to test the success of the viral vectors each experimental group was n = 3. For the preparation of brain slices, animals were euthanized by cervical vertebrae dislocation followed by decapitation. Further fixation was performed without preliminary perfusion, and all manipulations were performed in a dimly lit place. The surgically removed brains were placed in 4% paraformaldehyde solution in PBS for 12 h at 4 °C. The samples were then transferred three times to PBS solution to remove excess fixative solution and placed in 15% sucrose for cryoprotection and incubated at 4 °C until saturation. Then, the solution was replaced with 30% sucrose. After saturation with cryoprotectant, brain samples were deep frozen for further preparation of 35 µm thick slices using a freezing-sliding microtome Leica GM 1520 (Leica, Wetzlar, Germany). The prepared slices were placed into 24-well plates containing PBS solution.

To enhance the signal of native eGFP, which is included in the used viral constructs, immunohistochemical analysis was performed using primary goat polyclonal anti-GFP antibodies (1:1000, Rockland Immunochemicals, Pottstown, PA, USA, 600-101-215), secondary donkey anti-goat IgG (H + L) cross-adsorbed secondary antibody, Alexa Fluor 488 (1:300, Invitrogen, Waltham, MA, USA, A-11055). DAPI (1:1000, Sigma-Aldrich, St. Louis, MO, USA, D9542) was used as a counterstain for the nucleus. Stained sections were washed with PBS then mounted with mounting medium on slides [9,52,53].

The sampling of brain tissues for assessment of morphological changes after hypoxia was carried out on days 1 and 7 after the modeling of acute hypobaric hypoxia. Slices preparation and fixation was carried out as described above. Blocking was performed with a solution of 0.5% Triton ×100, 0.5% Tween 20, and 5% FCS in PBS for 1 h. Immunohistochemical labeling of MAP2, NeuN, GFAP was carried out using monoclonal antibodies Rabbit Anti-MAP2, Mouse Anti-NeuN, Chicken Anti-GFAP (ab183830, ab104224, ab4674, respectively, Abcam, Cambridge, UK) at a dilution of 1:500; incubation with primary antibodies lasted 24 h. Secondary antibodies Alexa Fluor^®^ 647 goat anti-rabbit, Alexa Fluor^®^ 488 Goat anti-Mouse, Alexa Fluor^®^ 546 Goat anti-Chicken (A21244, A10680, A11040, respectively, Thermofisher, Waltham, MA, USA) were used at a dilution of 1:1000; incubation lasted 1.5 h. Samples were mounted using Fluoroshield Mounting Medium with DAPI (ab104140, Abcam, Cambridge, UK).

A laser scanning microscope LSM 800, Plan-Apochromat 10×/0.3 objective (Zeiss, Oberkochen, Germany) was used for the imaging.

### 4.5. RNA Extraction and RT-PCR

Real-time PCR was used to assess the expression levels of neurotrophins (BDNF and GDNF) and their receptors (TrkB and GFRa1). Total RNA from the brains of one-month-old mice (cerebral cortex and hippocampus) was extracted using ExtractRNA reagent (Eurogen, Moscow, Russia) according to the manufacturer’s protocol. The amount of isolated RNA was determined using UV spectrophotometry (Nanodrop One, ThermoFisher Scientific, Waltham, MA, USA). MMLV RT kit and random primer (Eurogen, Moscow, Russia) was used for reverse transcription.

The following sequences of primer pairs were used:

Oaz1_fw 5′-AAGGACAGTTTTGCAGCTCTCC-3′, Oaz1_rv 5′-TCTGTCCTCACGGTTCTTGGG-3′;

BDNF_fw 5′-CCCAACGAAGAAAACCATAAGGA-3’, BDNF_rv 5’-CCAGCAGAAAGAGTAGAGGAGGCT-3′;

TrkB_fw 5′-TTTCCGCCACCTTGACTTGTCT-3′, TrkB_rv 5′-GTCGGGGCTGGATTTAGTCTCC-3′;

GDNF_fw 5′-CCTTCGCGCTGACCAGTGACT-3′, GDNF_rv 5′-GCCGCTTGTTTATCTGGTGACC-3′;

GFRα1_fw 5′-TGTCTTTCTGATAATGATTACGGA-3′, GFRα1_rv 5′-CTACGATGTTTCTGCCAATGATA-3′.

qPCR conditions: 50 °C for 2 min, 95 °C for 10 min, 40 cycles of 95 °C for 15 s, 60 °C for 60 s on an Applied Biosystems 7500 thermocycler (Applied Biosystems, ThermoFisher Scientific, Waltham, MA, USA) using the reaction mixture qPCRmix-HS SYBR + LowROX (Eurogen, Moscow, Russia).

Data were processed using the ΔΔCt method and a control sample (intact mouse without injections) in which the expression level was taken as one. Oaz1 was used as a housekeeping gene to normalize the data obtained. One of the most important characteristics of a reference gene candidate is a stable expression, i.e., a gene with a small coefficient of variation (CV) and a maximum fold change <2 (MFC, the ratio of the maximum and minimum values observed within the dataset). In addition, a mean expression level lower than the maximum expression level subtracted with 2 standard deviations (SD) was a prerequisite for a candidate housekeeping gene.

Based on these requirements, we selected the Oaz1 gene with the following parameters: 0.45 (SD), 3.78 (CV, %), and 1.51 (MFC). For a visual comparison: GAPDH is 0.74 (SD), 5.75 (CV, %), and 6.37 (MFC) [54].

### 4.6. Neurological Status Analysis

When evaluating the neurological status according to scale for assessing the neurological deficit in small laboratory animals, 10 involuntary congenital behavioral reactions (test of the animal’s spontaneous activity, symmetry in the movement of the four paws, proprioception of the animal’s body, reaction to touching vibrissae, ability to climb the net, test of pulling up on the bar, etc.) are tested. Each of them is scored from zero to two points, where two points mean no reaction. Interpretation of the results of neurological status scale evaluation: 10–20 points, severe CNS damage; 6–9 points moderate CNS damage; 1–5 points, mild CNS damage.

The Garcia scale uses 6 tests to assess the asymmetry of the animal’s movements and reactions in points (from 1, pronounced impairment, to 3, no impairment), which are also then summarized. The minimum neurological result is 3 points (maximum impairment), and the maximum is 18 points (no impairment) [55].

### 4.7. Acute Hypobaric Hypoxia Model

Acute hypobaric hypoxia (AHH) was modeled on the 30th day after the injection of viral vectors (Figure 10) according to the protocol described in [56]. In hypoxia modelling experiments the animals were divided into 6 groups: “Intact”–non-treated mice (no AHH modeling), n = 6; “Hypoxia”–only AHH modeling, no injection n = 21; “Hypoxia + PBS”–animals with sodium phosphate-buffered saline (PBS) intraventricular injection and AHH modeling, n = 35; “Hypoxia + AAV-Syn-eGFP”–animals with AAV-Syn-eGFP injection and AHH modeling, n = 18; “Hypoxia + AAV-Syn-BDNF-eGFP”–animals with AAV-Syn-BDNF-eGFP injection and AHH modeling, n = 22; “Hypoxia + AAV-Syn-GDNF-eGFP”–animals with AAV-Syn-GDNF-eGFP injection and AHH modeling, n = 18. 

Experimental animals were placed in a hypobaric chamber with the conditions corresponding to an ascent to an altitude of 10,000–10,500 m (170–185 mm Hg) at a speed of 183 m/s. The animals remained at this height until the second agonal breath, after which they were taken out from the pressure chamber. If the agonal breath did not occur within 10 min, the animal was taken out from the pressure chamber. The time from the moment of ascending to the height until the second agonal breath was estimated as the survival time. Animals whose lifetime at height was less than 3 min were categorized as low resistant to hypoxia, from 3 to 7 min as medium resistant, and more than 7 min as highly resistant. Then, the animals were returned to the house cage, where they were kept for a month. Male C58BL/6 mice were used in the study. Females were excluded from it because acute hypobaric hypoxia was simulated at P30. At the age of 28–35 days, puberty occurs in female mice; significant hormonal changes with the further establishment of a regular cycle are observed in animals [57,58]. The study design included behavioral tests (Morris water maze), so females were not used in the study to exclude the effect of hormonal changes on learning and recall of memory traces. The use of male mice in studies of rodent resistance to hypoxic and ischemic damage, including behavioral testing, is the gold standard [41,59,60,61]. Mice were housed in groups of 5–7 animals.

Therefore, our study had some limitations. First, we assessed the effect of BDNF and GDNF overexpression on resistance to hypoxic damage only in male mice. Further studies will be necessary to assess the potential gender-specific features of the influence of overexpression of these neurotrophins. Second, we injected a viral construct to induce the overexpression of neurotrophic factors in neurons in newborn animals, i.e., 30 days before hypoxia modeling. This approach cannot be directly used as a method of therapy. However, it is this protocol that makes it possible to simulate a damaging factor during the period when a stable increased expression of neurotrophic factors is established and to study the fundamental mechanisms of their neuroprotective action and their role in the individual adaptive capabilities of the organism. It is also important to note that we used an intraventricular injection of a viral vector, which allows to induce overexpression of the gene of interest not in a narrowly localized area, but in a large area of the brain.

### 4.8. Morris Water Maze

To assess cognitive and mnestic functions in the late post-hypoxic period, experimental animals were tested using the Morris water maze 1 month after modeling AHH. The animals who survived after hypoxia modelling were used in the cognitive testing post-hypoxia experiment. The “Intact” group was not exposed to hypoxia when tested in the Morris water maze.

The test protocol consisted of five training sessions, three attempts each. Training was conducted daily at the same time. During the experiment, the animal was placed to the maze in 3 different locations in relation to platform. The platform location was not changed during the test. Each attempt lasted 60 s. In case of platform found, the animal stands on the platform for 15 s. Then, 24 h after the last training session, animals were tested for long-term memory retention. The test lasted 60 s, during which there was no platform in the maze. The delayed coefficient of long-term memory retention (dCr) was determined as a ratio of the time spent by the mouse in the quadrant with a platform to the total time spent by the mouse in the Morris water maze.

Besides, the strategy of finding a platform during the test swim was evaluated. Three search strategies were identified: direct search, where the animal was going directly to the place of the platform’s former location; active search, where the animal carried out circular and radial search movements before reaching the target; chaotic search, where the absence of a clear strategy for reaching the target [62].

### 4.9. Statistical Analysis

The data obtained are presented in the format mean ± standard error of the mean (M ± SEM). ANOVA test and post-hoc Dunnett’s test were used for analysis of animals’ survival and resistance to hypoxia. The Kruskal–Wallis test by ranks was used for the analysis of Morris water maze data that differ from a normal distribution. The differences were considered significant in the case of a significance level *p* ≤ 0.05. The significance of differences between the experimental groups was determined using the SigmaPlot 11.0 program (Systat Software Inc., Richmond, CA, USA).

## 5. Conclusions

It is assumed that overexpression of the neurotrophic factors BDNF and GDNF through viral constructs can be considered an effective method of neuroprotection in hypoxic states.

## Figures and Tables

**Table 1:** Evaluation of neurological status of male C57BL/6 mice 30 days after AAV-Syn-BDNF-eGFP and AAV-Syn-GDNF-eGFP transduction.

Group of Animals	Neurological Deficit Assessment Scale	Garcia Scale
Intact (n = 24)	1.1 ± 0.4	17.1 ± 0.8
AAV-Syn-BDNF-eGFP (n = 25)	1.4 ± 0.3	17.3 ± 0.5
AAV-Syn-GDNF-eGFP (n = 21)	1.3 ± 0.4	17.5 ± 0.7

no statistically significant differences between groups.

## Data Availability

The data that support the findings of this study are available from the corresponding author upon reasonable request.
